# Assessment of the effect of larval source management and house improvement on malaria transmission when added to standard malaria control strategies in southern Malawi: study protocol for a cluster-randomised controlled trial

**DOI:** 10.1186/s12879-017-2749-2

**Published:** 2017-09-22

**Authors:** Robert S. McCann, Henk van den Berg, Peter J. Diggle, Michèle van Vugt, Dianne J. Terlouw, Kamija S. Phiri, Aurelio Di Pasquale, Nicolas Maire, Steven Gowelo, Monicah M. Mburu, Alinune N. Kabaghe, Themba Mzilahowa, Michael G. Chipeta, Willem Takken

**Affiliations:** 1 Wageningen University and Research, Wageningen, The Netherlands; 2 College of Medicine, University of Malawi, Blantyre, Malawi; 3 Lancaster University, Lancaster, UK; 4 Academic Medical Centre, University of Amsterdam, Amsterdam, The Netherlands; 5 Liverpool School of Tropical Medicine, Liverpool, UK; 6 Malawi-Liverpool Wellcome Trust, Blantyre, Malawi; 7 Department of Epidemiology and Public Health, Swiss Tropical and Public Health Institute, Basel, Switzerland; 8 University of Basel, Basel, Switzerland; 9 Laboratory of Entomology, Wageningen University and Research, PO Box 16, 6700 AA Wageningen, The Netherlands

**Keywords:** *Anopheles* mosquitoes, Integrated vector management, Larval source management, House improvement, Vector control, Malaria transmission, Community participation

## Abstract

**Background:**

Due to outdoor and residual transmission and insecticide resistance, long-lasting insecticidal nets (LLINs) and indoor residual spraying (IRS) will be insufficient as stand-alone malaria vector control interventions in many settings as programmes shift toward malaria elimination. Combining additional vector control interventions as part of an integrated strategy would potentially overcome these challenges. Larval source management (LSM) and structural house improvements (HI) are appealing as additional components of an integrated vector management plan because of their long histories of use, evidence on effectiveness in appropriate settings, and unique modes of action compared to LLINs and IRS. Implementation of LSM and HI through a community-based approach could provide a path for rolling-out these interventions sustainably and on a large scale.

**Methods/design:**

We will implement community-based LSM and HI, as additional interventions to the current national malaria control strategies, using a randomised block, 2 × 2 factorial, cluster-randomised design in rural, southern Malawi. These interventions will be continued for two years. The trial catchment area covers about 25,000 people living in 65 villages. Community participation is encouraged by training community volunteers as health animators, and supporting the organisation of village-level committees in collaboration with The Hunger Project, a non-governmental organisation. Household-level cross-sectional surveys, including parasitological and entomological sampling, will be conducted on a rolling, 2-monthly schedule to measure outcomes over two years (2016 to 2018). Coverage of LSM and HI will also be assessed throughout the trial area.

**Discussion:**

Combining LSM and/or HI together with the interventions currently implemented by the Malawi National Malaria Control Programme is anticipated to reduce malaria transmission below the level reached by current interventions alone. Implementation of LSM and HI through a community-based approach provides an opportunity for optimum adaptation to the local ecological and social setting, and enhances the potential for sustainability.

**Trial Registration:**

Registered with The Pan African Clinical Trials Registry on 3 March 2016, trial number PACTR201604001501493.

**Electronic supplementary material:**

The online version of this article (10.1186/s12879-017-2749-2) contains supplementary material, which is available to authorized users.

## Background

The main interventions currently recommended and used for malaria control in endemic regions are diagnosis and treatment with effective drugs (e.g. artemisinin-based combination therapies; ACTs), preventative therapy (e.g. intermittent preventative therapy in pregnant women; IPTp), and vector control with insecticide-treated nets (ITNs) and indoor residual spraying (IRS). These interventions have been clearly documented to be effective at reducing malaria burden when population-level coverage and use are high [1–4]. The halving of *Plasmodium falciparum* prevalence and the prevention of around 600 million clinical cases of malaria in endemic regions of Africa between 2000 and 2015 has been attributed to the mass implementation of ACTs, ITNs and/or IRS [5]. Vector control contributed to more than 70% of this reduction, demonstrating the importance of interrupting mosquito-human contact in the fight against malaria.

Despite these tremendous gains at controlling malaria in both low- and high-transmission settings, there are a number of well-documented challenges limiting the continued impact of the current interventions. Achieving and sustaining high access and use has proven difficult in most settings [6-8] due to inadequate infrastructure [9–11], lack of funding [8, 11, 12], durability and attrition in the case of ITNs [13, 14], and potential barriers related to knowledge, behaviour and/or attitudes of the target population [15–18]. Even with sustained high coverage, currently-recommended interventions alone are unlikely to eliminate malaria in high transmission settings [19, 20], or where part of the mosquito vector population bites or rests outdoors [21–24]. The development of resistance to anti-malaria drugs and insecticides by malaria parasite populations [25, 26] and vector populations [27, 28], respectively, poses yet further challenges.

The global community therefore continues to search for new, innovative solutions for malaria control [29]. One approach getting more attention over the past decade is the integration of multiple interventions, targeting the disease from multiple directions and therefore potentially leading to additive or synergistic effects beyond that of any single method on its own [30–33]. Integrated methods are well established for the control of insects, weeds and diseases in agriculture [34], providing a reliable framework for the development of integrated control programmes in public health [35]. Combining multiple methods has the additional, significant benefit of delaying the development of drug resistance [36] and insecticide resistance if applied as part of a resistance management strategy [37].

Two methods that could be integrated into existing National Malaria Control Programme (NMCP) strategies are larval source management (LSM) and structural house improvement (HI). Larval source management is commonly defined as the management of water bodies that are potential larval habitats of mosquitoes to prevent the completion of development of the immature stages [38–40]. Generally, there are four types of LSM: habitat modification (a permanent alteration to the environment such as land reclamation); habitat manipulation (a recurrent activity such as flushing of streams); larviciding (the regular application of biological or chemical insecticides to water bodies); and biological control (the introduction of natural predators into water bodies) [40]. LSM was part of successful malaria control campaigns in the United States, Israel, and Italy [38], and integral to eliminating the African malaria vector, *Anopheles gambiae*, from Brazil and Egypt [40]. In more recent controlled trials where LSM has been the primary intervention, it has reduced malaria prevalence in some, but not all, settings [39]. Indeed, knowledge about the ecology of local malaria vector populations and implementation of LSM methods deemed suitable for the specific setting are two critical components for a successful LSM programme [41].

Most currently available methods of LSM carry very little or no risk of reduced effectiveness over time due to insecticide resistance. No insecticides are used for habitat modification, habitat manipulation, and biological control. The most common larvicides use bacteria of the genus *Bacillus*, which produce multiple mosquito-specific toxins [42]. The use of multiple toxins at once reduces the selective pressure for insecticide resistance, and there is very little evidence of resistance developing in natural mosquito populations despite wide-spread use of *Bacillus thuringiensis israelensis* (*Bti*) in the United States of America, Europe, and elsewhere [42, 43]. If used as part of an integrated control programme, the different biological mode of action for *Bti*, compared with insecticides used in ITNs and IRS, further limits the risk of mosquitoes developing resistance to *Bacillus*-based larvicides. Furthermore, LSM targets both outdoor and indoor biting/resting mosquitoes equally (because it targets immature stages), a critical issue for addressing residual transmission [44–47].

Improved housing also has a long history of association with reducing malaria transmission [48]. In regions where malaria vectors bite indoors, improved housing reduces malaria transmission by reducing mosquito entry into houses [49–51]. Methods of house improvement include plastering walls and ceilings, filling crevices and holes, and screening eaves and windows of houses or sleeping quarters [48, 51–53]. Improved housing resulting from more favourable social and economic conditions is associated with less malaria, even when reducing mosquito entry is not necessarily the primary goal of the housing design [54, 55]. House improvement as an intentional intervention has been shown to reduce the number of *Anopheles* mosquitoes indoors [51, 56], and reduce the risk of anaemia in children [51]. As a malaria control intervention, house improvement relies on a mechanical/physical barrier, and does not, in principle, require any insecticide to reduce house entry by mosquitoes. As a physical barrier to reduce mosquito-human contact, it has the advantage of being less dependent on individual use/behaviour once it is in place. A further advantage is that it can protect everyone inside a “treated” house.

Achieving high community coverage is a challenge for currently-implemented and potential additional interventions alike. Focusing on community participation in public health efforts is one approach for addressing this challenge [57, 58]. Active community participation can increase local programme ownership, encourage awareness of health promotion, increase uptake of interventions, and ultimately lead to reduced disease incidence or prevalence [58–61]. Behaviour change communication strategies can be an essential component of community participation when introducing new interventions or scaling-up current interventions. Vector control programmes, in particular, have a high potential for success when community participation is explicitly incorporated [32, 62–65].

Because LSM and HI target mosquitoes through “modes of action” that are entirely different from ITNs and IRS, these two interventions have a high potential to complement existing intervention methods as part of an integrated malaria control programme and lead to additional reductions in malaria transmission. Considering this potential, we designed the trial described here with the primary objective to determine the impacts of HI and LSM on malaria parasite prevalence and entomological inoculation rate (EIR) over a 24-month period, when implemented alone or in combination, in addition to the Malawi NMCP interventions. We explicitly assume that NMCP interventions are implemented at the Scale-up for Impact coverage targets [66], and we explicitly implement both LSM and HI through a community-based approach. We postulate the use of LSM and/or HI in settings with high ITN and ACT use will lead to additional reductions in malaria transmission and burden on top of the effects from ITN and ACT use. The design of the trial does not allow for testing the potential for either LSM or HI to be used as a stand-alone strategy, nor are we directly comparing the effect of LSM or HI alone to the effect of ITN and ACT use.

## Methods/Design

### Study site

The study site is in Chikhwawa District, an area of high malaria transmission in the Lower Shire River Valley region of southern Malawi [67]. Chikhwawa is a mainly rural area with a population of over 530,000 people covering an area of about 4800 km^2^. Rain-fed farming is the main occupation, with maize, millet and sorghum as the major staple foods. Three malaria vector species are present: *Anopheles gambiae* s.s., *Anopheles arabiensis*, and *Anopheles funestus* [68]. Malaria control in Chikhwawa follows guidelines set by the NMCP, and as of 2015 relied on the use of ITNs (specifically, long-lasting insecticidal nets; LLINs) and IPTp for prevention, microscopy or rapid diagnostic tests (RDT) for diagnosis, and ACTs for treatment. Brands of LLIN distributed by the NMCP include PermaNet® 2.0 (Vestergaard Frandsen, Lausanne, Switzerland), Olyset® Net (Sumitomo Chemical Company, Tokyo, Japan), and as of 2016, Royal Sentry® (Disease Control Technologies, USA). The coverage targets for these interventions, as defined by the NMCP, are listed in Table 1. Poor roads and other infrastructure are just some of the challenges faced by the Chikhwawa District Health Office (DHO) in meeting these goals. The NMCP implemented IRS in Chikhwawa in 2010 and 2012 with alphacypermethrin [69]. Indoor residual spraying is not planned for the study site during the trial period. Resistance to the pyrethroids used in LLINs (permethrin and deltamethrin) has been reported for populations of*An. funestus*, and to a lower extent, *An. gambiae* s.l., in Chikhwawa [69–71].

**Table 1:** Description of Malawi NMCP 2011–2015 SUFI targets [66]

Indicator	Target
ITN ownership	90% of households own at least 1 ITN
ITN use by pregnant women	80% of pregnant women sleep under an ITN
ITN use by CU5	80% of CU5 sleep under ITN
IPTp	80% of pregnant women receive 2 or more doses of SP during pregnancy for malaria prevention
Case management	50% of suspected malaria cases at health care facilities confirmed by microscopy
Case management	80% of suspected malaria cases at health care facilities confirmed by RDT
Case management	50% of confirmed malaria cases appropriately treated within 24 h of onset of symptoms

*CU5* children under 5 years of age, *ITN* insecticide-treated net, *IPTp* intermittent preventative therapy in pregnant women, *NMCP* National Malaria Control Programme, *RDT* rapid diagnostic test, *SUFI* scale-up for impact

The trial villages are all located within the catchment area of the Majete Malaria Project (MMP), a collaboration of the Ministry of Health, The Hunger Project (THP; a non-governmental organisation specialising in community-based programmes), African Parks-Malawi (which has run the Majete Wildlife Reserve as part of a public-private partnership since 2003), and the academic institutions of the principal investigators of this trial. The project aims to reduce the malaria burden of the people living within about 10 km of Majete Wildlife Reserve, indirectly serving conservation interests by proposing that through improved health and socio-economic conditions, the community will become a better partner in natural resource management. The project considers community engagement and participation as a central focus of its strategy and follows THP’s principles to implement behaviour change communication and vector control programmes through a community-based approach.

**Fig. 1:**
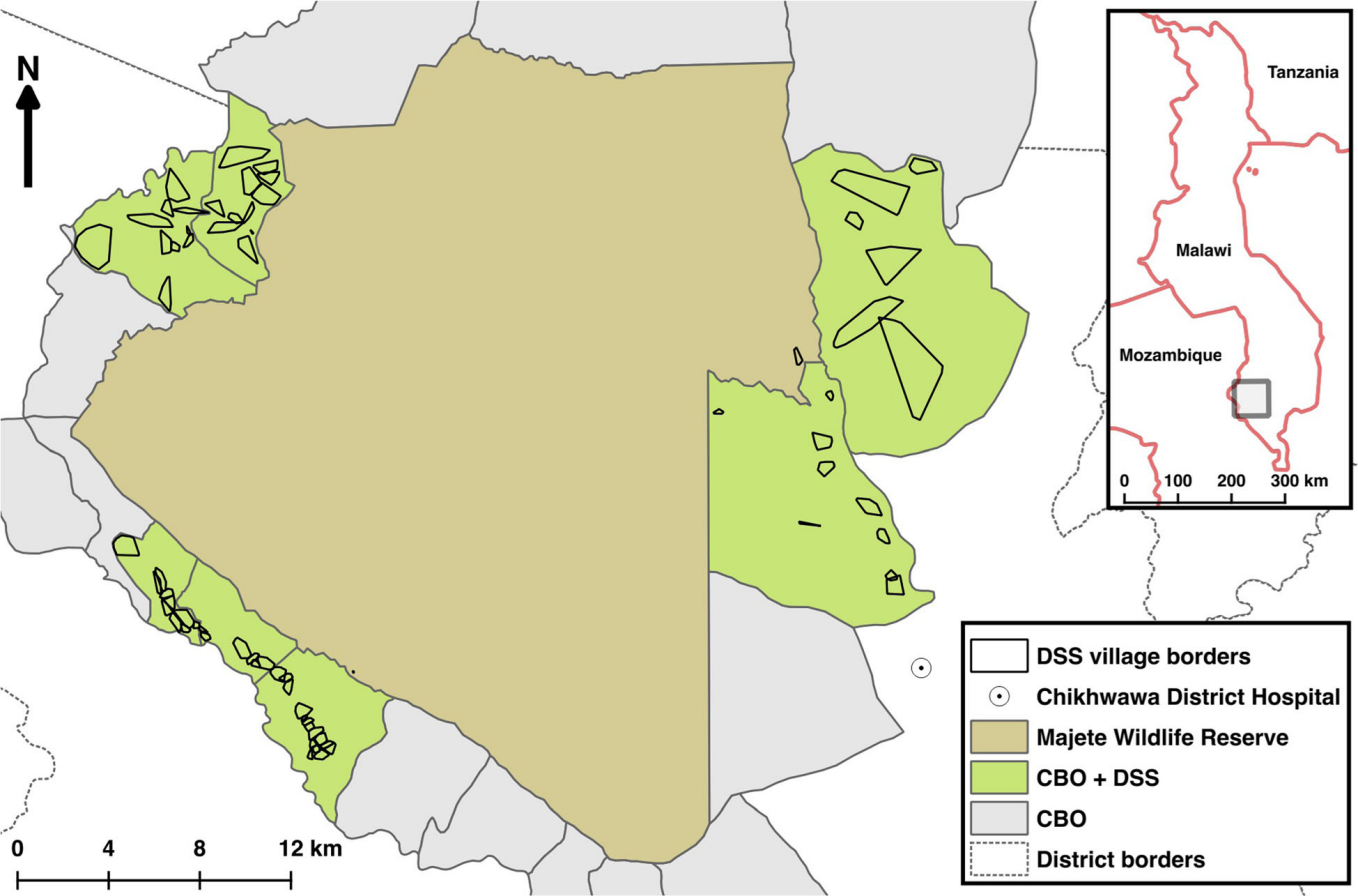
Study site map. Majete Wildlife Reserve, surrounded by 19 groups of villages known as community-based organisations (CBO). Trial villages fall under 7 of these CBOs, representing the 3 focal areas, or blocks. All villages in these 7 CBOs were enumerated into a demographic surveillance system (DSS). Reprinted with slight modification from Kabaghe et al. [90]

Since starting in 2014, MMP has initially concentrated efforts in three regions, which we refer to as focal areas A, B and C, respectively (Fig. 1). The three focal areas are spaced roughly evenly around the wildlife reserve and cover a total population of about 25,000 people in 65 villages. Focal areas were delineated to cover the same villages as an existing or planned THP *Epicentre*, which brings together neighbouring villages as a basis for community-led development [72].

The health system serving the area consists of 1 district referral hospital, 1 mission hospital, 1 private health centre, 6 government health centres, 6 health posts and 12 village clinics.

We conducted an enumeration from August 2014 through February 2015, whereby for every household we collected data on the name, gender, date of birth and relationship to the head of household for each member of the household. Here we defined a household as “a social group made up of people eating from the same pot”. Global positioning satellite (GPS) devices were used to record the geo-location of each house (structure) in which people slept at night, whereby the members of a household (social group) could be split among multiple houses (structure). Each house was given a unique identification code, and a pre-printed label showing the code was adhered to the door of the house immediately following the survey. Data were electronically collected and managed using a combination of Open Data Kit (ODK) and OpenHDS software [73].

### Community sensitisation and engagement

As implementing partners of MMP, staff from THP, College of Medicine, African Parks, and the DHO were responsible for sensitising the community about MMP in general, and the trial specifically. Accordingly, meetings were held separately at the District, Traditional Authority, Group-Village, and Village administrative levels to engage community leaders and stakeholders and sensitise them about MMP and the upcoming trial. From there, community engagement was an on-going process involving discussions between MMP staff, community leaders, and community members at the focal area and village levels.

To encourage the use of existing, NMCP malaria interventions, and to set the foundation for the community-based implementation of the trial interventions, an “animator approach” adapted to the specific setting was used. Volunteers from the 65 villages (slightly more than one per village on average) were trained as “health animators” by MMP, and thereafter these health animators led fortnightly malaria workshops in their communities. An essential component of this approach is empowering the community through a process of mindset change, leadership, vision, commitment and action [74]. In brief, this means that the community should perceive malaria as a challenge that can be actively addressed, and it provides a basis for community action planning towards malaria control. Furthermore, health animators followed a training manual, developed by the project, to cover a broad range of malaria topics at each of the community workshops. Following this approach, we expected that use of NMCP malaria interventions was equal across all villages in the trial. Still, this will be monitored via household surveys, as explained below.

### Eligibility

**Fig. 2:**
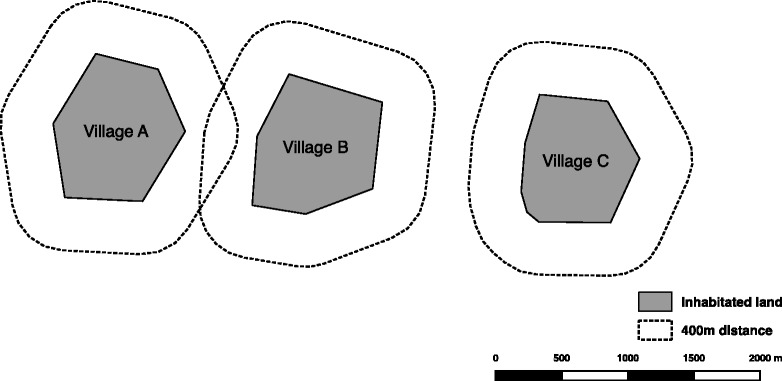
Example showing how buffer zones were used to define contamination distance. Village A and Village B could not be assigned to different treatments without risking contamination bias. Village C is assumed to be a sufficient distance from Villages A and B to limit contamination bias

Using village as the unit of randomisation, all households in the 65 enumerated villages were initially eligible for coverage with the trial interventions, in principle. Recognising the need for villages with different trial-treatments to have some minimum distance separating them to reduce the risk of contamination bias due to mosquito flight [75], we randomly excluded some of the eligible villages from the trial using the following approach. The objective of the approach was to identify a set of villages within each enumerated focal area to include in the trial that: 1) minimised contamination bias; 2) minimised selection bias; 3) maximised statistical power; and 4) gave every village at least one chance to be included so as to maximise perceived fairness by the participating communities. Within each focal area, we started with a map of the enumerated villages and overlaid 400 m buffer zones (Fig. 2) to identify which villages violated our a priori definition of contamination distance (i.e. any overlap of the 400 m buffer zones surrounding each possible pair of 2 villages). The distance of 400 m was determined after considering current knowledge about dispersal of the dominant malaria vector mosquito species in Africa [76]. While the maximum dispersal of *Anopheles* mosquitoes may be up to several kilometres under certain conditions, we also accounted for the relatively high human population density of our study area and assumed that the majority of *Anopheles* mosquitoes here would disperse no more than a few hundred metres.

**Fig. 3:**
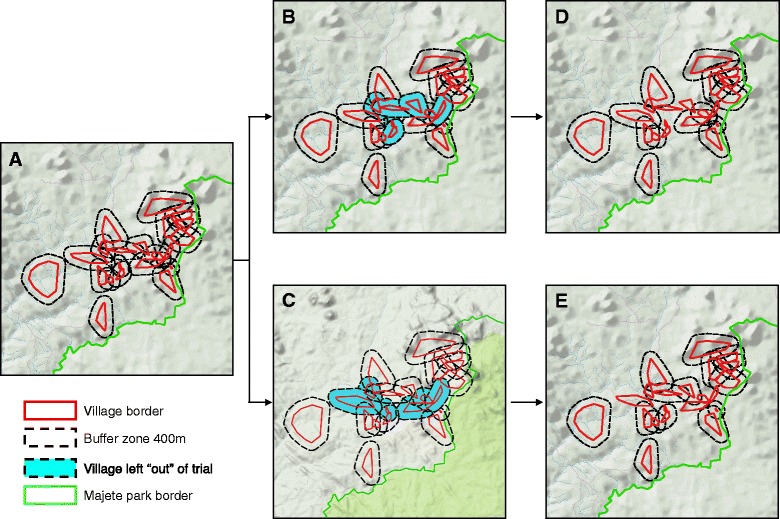
Random exclusion of villages. All five maps show the 21 villages of the focal area to the west of Majete Wildlife Reserve. **a** shows a 400 m buffer overlaid on each of the 21 villages. Nineteen of the villages have overlapping buffers, while two villages are a sufficient distance from the others to limit contamination bias. **b** and **c** highlight two different sets of villages that could be excluded from the trial intervention allocation, so as to leave the clusters of villages shown in **d** and **e**, whereby clusters do not overlap with each other

**Fig. 4:**
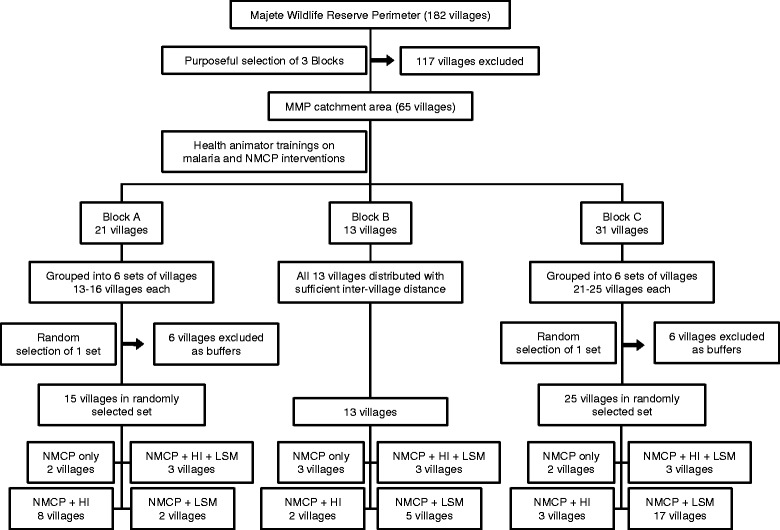
Flow chart showing the allocation of treatments to villages. HI, house improvement; LSM, larval source management; NMCP, National Malaria Control Programme

The maps of villages with overlaid buffer zones gave us an initial set of *clusters*, which were defined as a set of villages ranging in number from 1 to *n*, where *n* is the number of villages with overlapping buffer zones. For example, in Fig. 3a, there are 3 clusters. Due to the “natural layout” of village borders in some focal areas, including all villages in the trial would have given fewer clusters (*N* = 3) of more villages (maximum *n* = 19) than desired for maximising statistical power. To address this, we asked the question, “which villages should be excluded from the trial to give the maximum number of clusters possible in each focal area?”; and we answered the question using a solution from the mathematical field of graphs (i.e. maximum statistical power with restriction to the focal area). Knowing the maximum possible number of clusters, N_max_, we “manually” found alternative sets of villages which provided N_max_-1 to N_max_ clusters, and where the final list of sets included every village in the focal area at least once (i.e. maximum perceived fairness by the participating communities; Figs. 3b-d). Finally, one set of villages was randomly chosen in each focal area as the first stage of a 2-stage community raffle drawing (i.e. to minimise selection bias; Fig. 4)

### Design

The trial follows a randomised block, 2 × 2 factorial design (Table 2), with the three focal areas covered by MMP forming three blocks. The unit of randomisation for allocation of trial arms is the village, though we require all villages in the same *cluster* (above) to be randomised to the same treatment. The trial will run for two years, starting from the mass distribution of LLINs completed by the NMCP and Chikhwawa District Health Office on 1 May 2016.

**Table 2:** Trial arms showing interventions

Arm	NMCP	LSM	HI
1	√		
2	√	√	
3	√		√
4	√	√	√

*HI* house improvements, *LSM* larval source management, *NMCP* National Malaria Control Programme. For a description of NMCP interventions and target coverage, see Table 1

### Randomisation

There were two stages for the randomisation within each focal area (Fig. 4 ), which took place at a community event in each focal area to increase perceived fairness and engagement on the part of the participating communities. In the first stage, a set of villages was randomly selected from among six sets of villages satisfying the inclusion criteria described above. The six sets were numbered from 1 to 6, six cards numbered 1 to 6 were placed in a dish, and a volunteer from the community blindly selected one card. The remaining sets of villages were discarded. In one focal area (B) this stage was not necessary because the set of all villages in the focal area satisfied the inclusion criteria. From the set of included villages, treatments were randomly allocated to clusters, so that villages within a single cluster were randomised to the same treatment. The four trial arms were numbered from 1 to 4, cards numbered 1 to 4 were placed in a dish, and for each cluster a representative of the community selected one card to determine the treatment assigned to the villages in that cluster.

### Interventions

There are two interventions being evaluated in this trial: LSM and HI. While LSM generally refers to four broad types of activities [40], in this trial it refers specifically to habitat modification and larviciding. In this setting habitat modification refers to either draining or filling water bodies with the aim of permanently eliminating *Anopheles* larval habitats in cases where this is feasible and the water is not used for a specific community purpose. Larviciding will be done on all remaining potential *Anopheles* larval habitats with *Bti*, which are bacteria that produce insecticidal crystal proteins that, when ingested by larvae, attack the gut lining causing cessation of feeding and subsequent mortality [42]. Using the same “health animator approach” described above, communities in LSM villages are being trained and engaged in the concepts and practice of LSM. Following the project-led training of the health animators, each LSM village formed a LSM committee of 10–12 members tasked with organising all LSM activities in the village. For habitat modification, LSM committees and community leaders encourage all community members to participate in draining and filling potential larval habitats, and no material inputs are provided by the project. Additional training on the application of *Bti* was provided by the project to all LSM committee members to implement the application of *Bti* in their respective villages. Equipment and material (VectoBac WDG, Valent Biosciences, Libertyville IL, USA) for *Bti* application are provided by the project.

House improvement in this trial refers to material modification of houses aimed at blocking entry of malaria vectors. Following discussions with communities, the agreed modifications consist of: closing all eaves (i.e. where a wall meets the overhang of the roof) using local material similar to that used to construct the house (i.e. bricks and extra mud for most houses); closing all holes in the wall not used for ventilation using the same materials used for closing eaves; covering windows and other openings used for ventilation with aluminium screens that allow airflow; and modifying doors so as to fully cover doorways when closed. Similar to LSM, communities in HI villages are being engaged in the concepts and practices of HI through health animators and village-level committees. The project trained health animators on these concepts and practices, and then villages formed their own HI committees to organise and follow up on HI activities. In general, HI committees, community leaders and health animators encouraged heads of households in HI villages to carry out any necessary improvements on their own houses. When household members were unable to improve their houses on their own, HI committees assisted them. Materials provided by the project for HI were aluminium screening (allocated to each household based on surface area to cover, and distributed and managed by HI committees) and a set of basic hand tools shared and managed by the HI committees.

The coverage of LSM and HI in this trial depends heavily on community engagement and participation because the study design explicitly calls for community implementation of the interventions. Strategies to maximise “adherence”, and thus coverage, include the animator approach described above, livelihood development programmes (e.g. microfinance) implemented by THP, and regular monitoring of coverage by project staff with follow-ups. The animator approach is expected to improve coverage through an increased understanding of malaria, including the causes and mechanisms of prevention for each intervention. Development programmes associated with THP’s epicentres may further increase adherence by improving community engagement with the project. Finally, project staff will monitor coverage and encourage community-led corrective measures.

### Data collection

Mosquitoes will be sampled from the houses of study participants using so-called Suna traps (Biogents AG, Regensburg, Germany). The Suna trap is an odour-baited mosquito trap recently developed to collect host-seeking *Anopheles* mosquitoes both indoors and outdoors [77]. The trap uses CO_2_ produced through a process of yeast and molasses fermentation [78] and a synthetic blend of chemicals found on human skin [79] to attract mosquitoes. The odour blend used is standardised, allowing for reliable comparisons among trapping locations both indoors and outdoors. Alternative sampling methods, such as Centers for Disease Control and Prevention (CDC) light traps or pyrethrum spray catch, are not designed for outdoor use. In the case of CDC light traps, standard procedures for sampling African malaria vectors require the trap to be placed next to a person sleeping under a bed net [80].

Field teams will set up Suna traps at the houses of study participants and collect the mosquitoes from the traps each morning after a night of sampling. All mosquitoes collected will be preserved using a desiccant and identified using standard morphological and molecular techniques. Sampling will be conducted at each selected house one night indoors, and one night outdoors. Field teams will also collect basic information about the household through a standardised form. This will include observations of the types of bed nets in the house, the use of any insecticides, and the presence of livestock.

Epidemiological data will be collected using a household survey adapted from the internationally standardised malaria indicator survey (MIS) tool [81]. This tool includes a core questionnaire covering demographic and socio-economic aspects of the household and household members, and an additional module covering malaria control intervention practices and morbidity indicators. With written informed consent, a core questionnaire at household and individual level will be completed. Coverage indicators such as ownership and reported use of ITNs will be collected, regardless of the age of the ITN users. Data will also be collected on IPTp coverage (for women aged 15–49 years) and treatment-seeking behaviour for fever and ACT use (for children aged 6–59 months and women aged 15–49 years). Burden indicators will include parasitaemia as measured by RDT (SD BIOLINE Malaria Ag P.f. HRP-II, Standard Diagnostics, Yongin-si, Republic of Korea) and anaemia using HemoCue® Hb 301 (HemoCue, Ängelholm, Sweden) in children aged 6–59 months and women aged 15–49 years.

For the epidemiological surveys and adult mosquito sampling, we will use a repeated cross-sectional survey sampling framework [82]. Every two months from May 2016 through April 2018, 270 households will be selected for the epidemiological survey using a randomized inhibitory spatial sampling procedure [83]. At the same time, 195 of those 270 households will be randomly selected for adult mosquito sampling. The lower number of households for mosquito sampling is necessary because mosquito traps will be set at each selected household for two nights, whereas the epidemiological survey requires one day per household. All households in the trial villages will be eligible for selection each round regardless of whether they were selected in a previous round. Data collection at the 270 households will be conducted over a six-week period, with the last two weeks of each round reserved for data cleaning.

To determine coverage of HI, the house-level observations during adult mosquito sampling will also include: roof type; wall type; window type; open eaves; number and size of openings; coverage of windows with aluminium screens; and condition of the door. To determine coverage of LSM, regular monitoring of LSM villages will be conducted to record the number of potential larval habitats (bodies of standing water) drained and filled by the community. Larval mosquito populations will be assessed in all trial villages through regular monitoring of potential larval habitats. Larval mosquitoes will be collected using a standardised area sampling method, where 300 ml dippers and plastic pipettes are used to collect all *Anopheles* larvae within a 0.5m^2^ sampling quadrat.

A mixture of qualitative and quantitative methods will be used to collect data on feasibility, acceptability and appropriateness of the community engagement methods used. Qualitative methods will include participant observations, focus group discussions and in-depth interviews. Quantitative methods will include a cross-sectional standardised survey to assess knowledge, attitudes and practices. Economic data will be collected using a societal perspective and an ingredients approach to assess the cost and cost-effectiveness of community-implemented LSM and HI.

Environmental data will also be collected. Weather stations (HOBO® U30 Station, Onset® Computer Corporation, Bourne MA, USA) are set up in each of the three focal areas to collect hourly recordings of temperature, humidity, rainfall, wind direction and speed, and soil moisture. Landscape data (e.g. soil type, topography, land use-land cover, and monthly normalised difference vegetation index) will be retrieved from publicly-available sources such as the United States Geological Survey (USGS) and the International Soil Reference and Information Centre (ISRIC).

### Outcomes

The primary outcome is the estimated entomological inoculation rate (EIR) at the end of the intervention period. The EIR is calculated as the product of the human biting rate (HBR) and the sporozoite rate (SR), where HBR is the number of bites from a malaria vector species per year and SR is the prevalence of the malaria parasite infective stage (sporozoite) in the local malaria vector population [84]. Because Suna traps target host-seeking mosquitoes, the abundance of female malaria vectors collected per trap-night can be assumed to be proportional to the number of bites per person per night. For assessment of the primary outcome: indoor and outdoor Suna trap samples will be pooled; HBR for a given village will be calculated as the mean number of bites per person per night, multiplied by 365; SR will be calculated as the number of sporozoite-positive malaria vectors divided by the total number of female malaria vectors collected in a given village; and EIR will be the product of HBR and SR. EIR will be calculated by species, and then summed across species to get the total EIR. For primary analysis of the primary outcome, we will assume every household to be fully covered by the interventions in the trial arm to which it was allocated (i.e. assignment of intervention status will be by intention-to-treat). Data on intervention coverage, collected as described above, will be used in secondary analyses of the primary outcome. Secondary outcomes are shown in Table 3.

**Table 3:** Outcomes of interest to be analysed

Outcome	Metric	Source of Data
Entomological inoculation rate (EIR)*	abundance of female malaria vectors collected per trap-night, multiplied by proportion^†^ positive for *Plasmodium falciparum* sporozoites; indoor-only, outdoor-only, and pooled*	Routine monitoring of adult mosquitoes
Malaria vector community composition	Ratio^†^ of *An. funestus* to *An. gambiae* to *An. arabiensis*	Routine monitoring of adult mosquitoes
Malaria vector human blood index (HBI)	Proportion^†^ of *Anopheles* with human blood in abdomen out of all blood-fed *Anopheles*	Resting mosquito collections
Peak malaria vector biting time	Time of day (starting hour to ending hour) when 80% of host-seeking malaria vectors collected	Human landing collections
Larval mosquito density	Number of 3rd instar, 4th instar, and pupae per metre of potential larval habitat	Routine monitoring of larval mosquitoes
Parasite prevalence in children aged 6–59 months	Proportion^†^ of RDT tests positive for *Plasmodium falciparum*	Malaria indicator surveys
Prevalence of anaemia in children aged 6–59 months	Proportion^†^ of anemia tests with Hb < 8.0	Malaria indicator surveys
Incidence of clinical malaria in children aged 6–59 months	Number of clinical malaria cases per child per year	Incidence study cohorts

*Primary outcome^†^Raw data for proportions will be stored as separate numbers in the database, with actual proportions calculated at time of analysis only; also applies to ratios.

### Data handling

Tablets will be used for electronic data collection in the field with entry screens in ODK Collect and OpenHDS. Following collection by the field team and verification by a data officer, data will be sent to a central server running ODK Aggregate and OpenHDS [73] and managed in respective databases. Database access will be password protected. The data collection will be managed by the researchers, working independently from the project funder and sponsor.

### Statistics

The study design described above resulted in the inclusion of 53 villages, distributed as 15, 13 and 25 in focal areas A, B and C, respectively, with considerable imbalance across treatment categories as shown in Table 4. For the primary analysis, the outcome measure for each village is the estimated EIR at the end of the intervention period. Based on evidence from the literature [68, 85, 86] we assume that in the control arm, EIR has a log-Normal distribution with mean and standard deviation approximately 45 and 14, respectively, giving an effective range of approximately 0 to 100. We further assume that a clinically effective intervention will be one that halves the mean EIR.

**Table 4:** Number of villages in each trial arm, by focal area

Focal area	Control	HI	LSM	HI + LSM	Total
A	2	8	2	3	15
B	3	2	5	3	13
C	2	3	17	3	25
Total	7	7	24	9	53

HI house improvements, *LSM* larval source management

The statistical model for the primary analysis will be a randomised block ANOVA allowing for block effects plus main effects for each factor and an interaction term. Because the design is necessarily unbalanced, the estimated effects of the different treatment factors will have different standard errors. Under standard assumptions, namely that villages generate statistically independent outcomes, the resulting power to detect a clinically significant main effect of HI, testing at the conventional 5% level, is 0.669. The associated standard error of the estimated effect size is 0.286. This implies that a 95% confidence interval for the relative reduction in EIR associated with a clinically significant main effect will likely extend from 0.285 to 0.876. For the main effect of LSM, the corresponding figures are 0.728, 0.265 and a range from 0.298 to 0.840. We have no information on the possible strength of correlation between results from villages within the same cluster. To guard against this, we will test for significance using a robust version of the ANOVA F-test that respects the restricted randomisation.

Secondary analysis will be conducted at the household level, to allow for estimation of household-level covariate effects. We will fit a generalized linear mixed effects model to each outcome variable, treating village within focal area as a random effect. Real-valued outcomes will be analysed under Normal distributional assumptions, after transformation if necessary. Discrete outcomes will be analysed using binomial or Poisson distributional assumptions for closed and open counts, respectively, and including a second-level random effect for households within villages to allow for over-dispersion. Hence, with *i* denoting village, *j* denoting household within village and *d*_*ij*_ a vector of household-level covariates, the linear predictor for the generalized linear model will take the form

$$ {d}_{ij}\ \beta +{U}_i+{V}_{ij} $$

where the *U*_*i*_ and *V*_*ij*_ are mutually independent, Normally distributed random variables with means zero and variances σ^*2*^ and τ^*2*^, respectively.

We will also conduct a spatio-temporal analysis of any outcome variable for which the mixed model analysis identifies an important component of variation at village and/or household level. This will replace the unstructured village and/or household level random effects, *U*_*i*_ and *V*_*ij*_ *,* by a spatio-temporally correlated stochastic processes *S(x,t)* where *x* and *t* denote location and time, respectively. It will also use spatially and/or temporally varying environmental covariates in addition to household-level covariates identified as important in the household-level mixed model analyses.

### Timetable

**Fig. 5:**
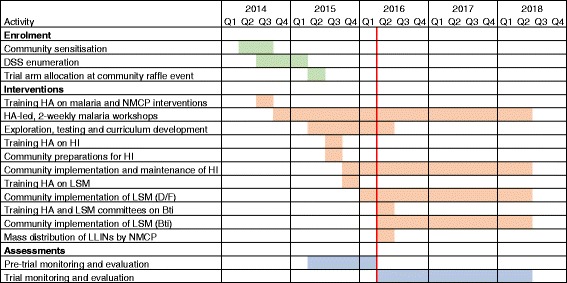
Trial timeline. The red line indicates the start of the trial. Bti *Bacillus thuringiensis israelensis*; DSS, demographic surveillance system; D/F, draining and filling; HA, health animators; HI, house improvement; LSM, larval source management; LLIN, Long-lasting insecticidal nets; NMCP, National Malaria Control Programme

Community engagement for the trial started in April 2014 (Fig. 5). Volunteers from all villages were trained as health animators in September and October 2014 and started leading fortnightly malaria workshops in their villages from November 2014, with plans to continue through to the end of the trial. A community raffle event was held in each focal area in June 2015 for allocation of villages to the trial arms. From April 2015 we worked with the community to develop locally applicable methods of LSM and HI, which were incorporated into training curricula for each of the interventions.

Health animators in HI villages were trained on HI in July 2015, and communities prepared for HI implementation through September 2015 by forming committees and preparing materials (e.g. bricks). Implementation of HI started in October 2015, with the aim of completing work before the rainy season started. Maintenance of HI will continue through the end of the trial. Health animators in LSM villages were trained on LSM in December 2015. Communities began implementing the habitat modification (draining and filling) part of LSM in January 2015, with continued maintenance planned through the end of the trial. Training on *Bti* for health animators and LSM committees was held in May 2016, with application of*Bti* starting the same month. The trial officially started on 1 May 2016 with the completion of a mass distribution of LLINs by the NMCP, ensuring high ownership of LLINs throughout the trial catchment area.

### Ethical considerations

This study complies with the principles described in the Declaration of Helsinki. This study was approved by the College of Medicine Research and Ethics Committee (COMREC) in Malawi (proposal number P.05/15/1731). Written permission to conduct the study was provided by the District Health Officer of Chikhwawa District. The WHO Trial Registration Data Set is given in Additional file 1. The SPIRIT protocol checklist is given in Additional file 2.

Using the community engagement structures described above, we informed the communities about the proposed trial activities and incorporated their feedback where appropriate. Community permission to conduct the trial was obtained from each village headman verbally, which was considered a culturally appropriate manner for the permission [87]. Community permission will cover all aspects of the trial, including the implementation of the proposed trial interventions and activities for assessment of trial outcomes. Specifically, community permission will be considered sufficient for proceeding with the interventions as described above. Individual informed consent will not be sought for the implementation of LSM and HI because the interventions are implemented at the community level. The project team has trained community members on the concepts and methods of LSM and HI, but the communities themselves will actually carry out the vector control activities. Further, the water bodies that will be affected by LSM generally belong to the community and not an individual, per se. Additionally, informed written consent will be sought from individual participants (and their parents or guardians if appropriate) during activities for assessment of entomological and epidemiological outcomes.

The larvicide to be used in this study, *Bti*, works through a very specific mode of action, making it unlikely to pose any hazard to humans or other vertebrates, or to the great majority of non-target invertebrates [88]. It has been shown previously to pose no risks to human health when used in aquatic habitats including drinking-water reservoirs [88].

## Discussion

While the use of ITNs and IRS for malaria vector control has considerably reduced the burden of malaria across the globe over the last 15 years, calls for eliminating malaria and management of insecticide resistance will require additional interventions to be implemented as part of integrated vector management strategies [89]. Particularly in settings where vector populations are resistant to pyrethroids, or bite outdoors or in the early evening, ITNs and IRS, which target only indoor-biting and indoor-resting mosquitoes, will likely be insufficient as stand-alone interventions to make sustained progress towards transmission reduction or to drive malaria transmission to zero [21, 22, 27]. Therefore, this trial is designed to measure the impact of two vector control interventions (LSM and HI) on malaria transmission and burden when implemented in addition to the Malawi NMCP interventions (LLINs, ACTs and IPTp). Combining LSM and/or HI together with the interventions currently implemented by the NMCP is anticipated to reduce malaria transmission below the level reached by current interventions alone. Both interventions could also be effective components of an insecticide resistance management programme by reducing the selective advantage of pyrethroid resistant individuals in the mosquito population.

Implementation of the two interventions, LSM and HI, is largely delegated to the community. Advantages of using this community-based as compared to researcher-based approach to implementation include: implementation can be done at a larger scale while still being tailored to the local setting; impact is being tested under more realistic, community-based conditions; and results will provide insight into the prospects for upscaling and sustainability.

This community-based approach to implementation is novel but depends heavily on the compliance of the community. As researchers, we aim to assure quality of the interventions through monitoring of community workshops and community activities, through evaluation of coverage indicators, and through introduction of corrective measures when and where necessary during the two-year trial. Corrective measures could include additional training for health animators and committee members, new training methods, use of local case studies, and instruction through monthly meetings of health animators and committee members.

We will keep records of these corrective measures, along with all inputs, processes, outputs and outcomes of community-based activities as essential components of the intervention implementation. We will also conduct socio-behavioural studies to assess the communities’ perceptions of the approach and economic studies to assess the costs and cost-effectiveness of this implementation approach.

## Additional files

Additional file 1: World Health Organization Trial Registration Data Set for the MMP LSM/HI trial. (DOCX 117 kb)

Additional file 2: SPIRIT checklist for the MMP LSM/HI trial, indicating which manuscript page contains each element of the study protocol. (DOC 123 kb)

## Abbreviations

ACT: Artemisinin-based combination therapies
Bti: Bacillus thuringiensis israelensis
CBO: Community-based organisations
CDC: Centers for Disease Control and Prevention
COMREC: College of Medicine Research and Ethics Committee
DHO: District Health Office
DSS: Demographic surveillance system
EIR: Entomological inoculation rate
GPS: Global positioning satellite
HBI: Human blood index;
HBR: Human biting rate
HI: House improvements
IPTp: Intermittent preventative therapy in pregnant women
IRS: Indoor residual spraying
ISRIC: International Soil Reference and Information Centre
ITN: Insecticide-treated net
LLIN: Long-lasting insecticidal net
LSM: Larval source management
MIS: Malaria indicator survey
MMP: Majete Malaria Project
NMCP: National Malaria Control Programme
ODK: Open Data Kit
RDT: Rapid diagnostic test
SR: Sporozoite rate
THP: The Hunger Project
USGS: United States Geological Survey

## References

[1] Lengeler C. Insecticide-treated bed nets and curtains for preventing malaria. Cochrane Db Syst Rev. 2004;(2):CD000363. 10.1002/14651858.CD000363.pub2 15106149

[2] Pluess B, Tanser FC, Lengeler C, Sharp BeL. Indoor residual spraying for preventing malaria. Cochrane Db Syst Rev. 2010;(4):CD006657. 10.1002/14651858.CD006657.pub2 PMC6532743 20393950

[3] Radeva-Petrova D, Kayentao K, Kuile ter FO. Drugs for preventing malaria in pregnant women in endemic areas: any drug regimen versus placebo or no treatment. Cochrane Db Syst Rev. 2014;(10):CD000169. 10.1002/14651858.CD000169.pub3 PMC4498495 25300703

[4] Sinclair D, Zani B, Donegan S, Olliaro P, Garner P. Artemisinin-based combination therapy for treating uncomplicated malaria. Cochrane Db Syst Rev. 2009;(3):CD007483. 10.1002/14651858.CD007483.pub2 PMC6532584 19588433

[5] Bhatt S, Weiss DJ, Cameron E, Bisanzio D, Mappin B, Dalrymple U (2015). The effect of malaria control on *Plasmodium falciparum* in Africa between 2000 and 2015. Nature.

[6] Wanzira H, Yeka A, Kigozi R, Rubahika D, Nasr S, Sserwanga A (2014). Long-lasting insecticide-treated bed net ownership and use among children under five years of age following a targeted distribution in central Uganda. Malar J.

[7] Koenker H, Kilian A (2014). Recalculating the net use gap: a multi-country comparison of ITN use versus ITN access. PLoS One.

[8] Bastiaens GJH, Bousema T, Leslie T (2014). Scale-up of malaria rapid diagnostic tests and artemisinin-based combination therapy: challenges and perspectives in Sub-Saharan Africa. PLoS Med.

[9] Larson PS, Mathanga DP, Campbell CH, Wilson ML (2012). Distance to health services influences insecticide-treated net possession and use among six to 59 month-old children in Malawi. Malar J.

[10] Theiss-Nyland K, Ejersa W, Karema C, Koné D, Koenker H, Cyaka Y (2016). Operational challenges to continuous LLIN distribution: a qualitative rapid assessment in four countries. Malar J.

[11] Chanda E, Doggale C, Pasquale H, Azairwe R, Baba S, Mnzava A (2013). Addressing malaria vector control challenges in South Sudan: proposed recommendations. Malar J.

[12] Chanda E, Mzilahowa T, Chipwanya J, Mulenga S, Ali D, Troell P (2015). Preventing malaria transmission by indoor residual spraying in Malawi: grappling with the challenge of uncertain sustainability. Malar J.

[13] Tan KR, Coleman J, Smith B, Hamainza B, Katebe-Sakala C, Kean C (2016). A longitudinal study of the durability of long-lasting insecticidal nets in Zambia. Malar J.

[14] Koenker H, Kilian A, de Beyl CZ, Onyefunafoa EO, Selby RA, Abeku T (2014). What happens to lost nets: a multi-country analysis of reasons for LLIN attrition using 14 household surveys in four countries. Malar J.

[15] Kateera F, Ingabire CM, Hakizimana E, Rulisa A, Karinda P, Grobusch MP (2015). Long-lasting insecticidal net source, ownership and use in the context of universal coverage: a household survey in eastern Rwanda. Malar J.

[16] Birhanu Z, Abebe L, Sudhakar M, Dissanayake G, Yihdego Y, Alemayehu G (2015). Access to and use gaps of insecticide-treated nets among communities in Jimma Zone, southwestern Ethiopia: baseline results from malaria education interventions. BMC Public Health.

[17] Strachan CE, Nuwa A, Muhangi D, Okui AP, Helinski MEH, Tibenderana JK (2016). What drives the consistent use of long-lasting insecticidal nets over time? A multi-method qualitative study in mid-western Uganda. Malar J.

[18] Chuma J, Okungu V, Molyneux C (2010). Barriers to prompt and effective malaria treatment among the poorest population in Kenya. Malar J.

[19] Griffin JT, Hollingsworth TD, Okell LC, Churcher TS, White M, Hinsley W (2010). Reducing *Plasmodium falciparum* malaria transmission in Africa: a model-based evaluation of intervention strategies. PLoS Med.

[20] Kleinschmidt I, Schwabe C, Benavente L, Torrez M, Ridl FC, Segura JL (2009). Marked increase in child survival after four years of intensive malaria control. Am J Trop Med Hyg.

[21] Bayoh MN, Mathias DK, Odiere MR, Mutuku FM, Kamau L, Gimnig JE (2010). *Anopheles gambiae*: historical population decline associated with regional distribution of insecticide-treated bed nets in western Nyanza Province, Kenya. Malar J.

[22] Russell TL, Govella NJ, Azizi S, Drakeley CJ, Kachur SP, Killeen GF (2011). Increased proportions of outdoor feeding among residual malaria vector populations following increased use of insecticide-treated nets in rural Tanzania. Malar J.

[23] Reddy MR, Overgaard HJ, Abaga S, Reddy VP, Caccone A, Kiszewski AE (2011). Outdoor host seeking behaviour of *Anopheles gambiae* mosquitoes following initiation of malaria vector control on Bioko Island, Equatorial Guinea. Malar J.

[24] Pates H, Curtis C (2005). Mosquito behavior and vector control. Annu Rev Entomol.

[25] White N (1999). Antimalarial drug resistance and combination chemotherapy. Philos Trans R Soc Lond B.

[26] Dondorp AM, Nosten F, Yi P, Das D, Phyo AP, Tarning J (2009). Artemisinin resistance in *Plasmodium falciparum* malaria. N Engl J Med.

[27] Ranson H, Lissenden N (2016). Insecticide resistance in African *Anopheles* mosquitoes: a worsening situation that needs urgent action to maintain malaria control. Trends Parasitol.

[28] Knox TB, Juma EO, Ochomo EO, Jamet HP, Ndungo L, Chege P (2014). An online tool for mapping insecticide resistance in major *Anopheles* vectors of human malaria parasites and review of resistance status for the Afrotropical region. Parasit Vectors.

[29] Takken W, Knols BGJ (2009). Malaria vector control: current and future strategies. Trends Parasitol.

[30] Protopopoff N, Wright A, West PA, Tigererwa R, Mosha FW, Kisinza W (2015). Combination of insecticide treated nets and indoor residual spraying in northern Tanzania provides additional reduction in vector population density and malaria transmission rates compared to insecticide treated nets alone: a randomised control trial. PLoS One.

[31] Gimnig JE, Otieno P, Were V, Marwanga D, Abong’o D, Wiegand R (2016). The effect of indoor residual spraying on the prevalence of malaria parasite infection, clinical malaria and anemia in an area of perennial transmission and moderate coverage of insecticide treated nets in western Kenya. PLoS One.

[32] Beier JC, Keating J, Githure JI, Macdonald MB, Impoinvil DE, Novak RJ (2008). Integrated vector management for malaria control. Malar J.

[33] Okia M, Okui P, Lugemwa M, Govere JM, Katamba V, Rwakimari JB (2016). Consolidating tactical planning and implementation frameworks for integrated vector management in Uganda. Malar J.

[34] Kogan M (1998). Integrated pest management: historical perspectives and contemporary developments. Annu Rev Entomol.

[35] Thomas MB, Godfray HCJ, Read AF, van den Berg H, Tabashnik BE, van Lenteren JC, et al. Lessons from agriculture for the sustainable management of malaria vectors. PLoS Med 2012;9:e1001262. 10.1371/journal.pmed.1001262 PMC3393651 22802742

[36] Boni MF, White NJ, Baird JK (2016). The community as the patient in malaria-endemic areas: preempting drug resistance with multiple first-line therapies. PLoS Med.

[37] World Health Organization (2012). Global plan for insecticide resistance management.

[38] Kitron U, Spielman A (1989). Suppression of transmission of malaria through source reduction: antianopheline measures applied in Israel, the United States, and Italy. Rev Infect Dis.

[39] Tusting LS, Thwing J, Sinclair D, Fillinger U, Gimnig JE, Bonner KE, et al. Mosquito larval source management for controlling malaria. Cochrane Db Syst Rev. 2013;(8):CD008923. 10.1002/14651858.CD008923.pub2 PMC4669681 23986463

[40] Fillinger U, Lindsay SW (2011). Larval source management for malaria control in Africa: myths and reality. Malar J.

[41] World Health Organization (2013). Larval source management: a supplementary measure for malaria vector control. An operational manual.

[42] Charles J-F, Nielsen-LeRoux C (2000). Mosquitocidal bacterial toxins: diversity, mode of action and resistance phenomena. Mem Inst Oswaldo Cruz.

[43] Becker N, Ludwig M (1993). Investigations on possible resistance in *Aedes vexans* field populations after a 10-year application of *Bacillus thuringiensis israelensis*. J Am Mosq Control Assoc.

[44] Meyers JI, Pathikonda S, Popkin-Hall ZR, Medeiros MC, Fuseini G, Matias A (2016). Increasing outdoor host-seeking in *Anopheles gambiae* over 6 years of vector control on Bioko Island. Malar J.

[45] Monroe A, Asamoah O, Lam Y, Koenker H, Psychas P, Lynch M (2015). Outdoor-sleeping and other night-time activities in northern Ghana: implications for residual transmission and malaria prevention. Malar J.

[46] Mwangangi JM, Muturi EJ, Muriu SM, Nzovu J, Midega JT, Mbogo CM (2013). The role of *Anopheles arabiensis* and *Anopheles coustani* in indoor and outdoor malaria transmission in Taveta District, Kenya. Parasit Vectors.

[47] Govella NJ, Ferguson HH (2012). Why use of interventions targeting outdoor biting mosquitoes will be necessary to achieve malaria elimination. Front Physiol.

[48] Lindsay SW, Emerson PM, Charlwood JD (2002). Reducing malaria by mosquito-proofing houses. Trends Parasitol.

[49] Kampango A, Bragança M, de Sousa B, Charlwood JD (2013). Netting barriers to prevent mosquito entry into houses in southern Mozambique: a pilot study. Malar J.

[50] Lindsay SW, Jawara M, Paine K, Pinder M, Walraven GEL, Emerson PM (2003). Changes in house design reduce exposure to malaria mosquitoes. Tropical Med Int Health.

[51] Kirby MJ, Ameh D, Bottomley C, Green C, Jawara M, Milligan PJ (2009). Effect of two different house screening interventions on exposure to malaria vectors and on anaemia in children in The Gambia: a randomised controlled trial. Lancet.

[52] Gunawardena DM, Wickremasinghe AR, Muthuwatta L, Weerasingha S, Rajakaruna J, Senanayaka T (1998). Malaria risk factors in an endemic region of Sri Lanka, and the impact and cost implications of risk factor-based interventions. Am J Trop Med Hyg.

[53] Ogoma SB, Kannady K, Sikulu M, Chaki PP, Govella NJ, Mukabana WR (2009). Window screening, ceilings and closed eaves as sustainable ways to control malaria in Dar es Salaam, Tanzania. Malar J.

[54] Tusting LS, Ippolito MM, Willey BA, Kleinschmidt I, Dorsey G, Gosling RD (2015). The evidence for improving housing to reduce malaria: a systematic review and meta-analysis. Malar J.

[55] Wanzirah H, Tusting LS, Arinaitwe E, Katureebe A, Maxwell K, Rek J (2015). Mind the gap: house structure and the risk of malaria in Uganda. PLoS One.

[56] Ogoma SB, Lweitoijera DW, Ngonyani H, Furer B, Russell TL, Mukabana WR (2010). Screening mosquito house entry points as a potential method for integrated control of endophagic filariasis, arbovirus and malaria vectors. PLoS Negl Trop Dis.

[57] Whittaker M, Smith C (2015). Reimagining malaria: five reasons to strengthen community engagement in the lead up to malaria elimination. Malar J.

[58] Atkinson J-A, Vallely A, Fitzgerald L, Whittaker M, Tanner M (2011). The architecture and effect of participation: a systematic review of community participation for communicable disease control and elimination, Implications for malaria elimination. Malar J.

[59] van den Berg H, Velayudhan R, Ebol A, Catbagan BHG, Turingan R, Tuso M (2012). Operational efficiency and sustainability of vector control of malaria and dengue: descriptive case studies from the Philippines. Malar J.

[60] Brieger WR (1996). Health education to promote community involvement in the control of tropical diseases. Acta Trop.

[61] Deressa W, Olana D, Chibsa S (2005). Community participation in malaria epidemic control in highland areas of southern Oromia, Ethiopia. Ethiop J Health Dev.

[62] van den Berg H (2007). Reducing vector-borne disease by empowering farmers in integrated vector management. Bull World Health Organ.

[63] Oria PA, Hiscox A, Alaii J, Ayugi M, Mukabana WR, Takken W (2014). Tracking the mutual shaping of the technical and social dimensions of solar-powered mosquito trapping systems (SMoTS) for malaria control on Rusinga Island, western Kenya. Parasit Vectors.

[64] Geissbühler Y, Kannady K, Chaki PP, Emidi B, Govella NJ, Mayagaya V (2009). Microbial larvicide application by a large-scale, community-based program reduces malaria infection prevalence in urban Dar Es Salaam, Tanzania. PLoS One.

[65] Opiyo P, Mukabana WR, Kiche I, Mathenge E, Killeen GF, Fillinger U (2007). An exploratory study of community factors relevant for participatory malaria control on Rusinga Island, western Kenya. Malar J.

[66] World Health Organization (2013). Focus on Malawi.

[67] Bennett A, Kazembe L, Mathanga DP, Kinyoki D, Ali D, Snow RW (2013). Mapping malaria transmission intensity in Malawi, 2000-2010. Am J Trop Med Hyg.

[68] Mzilahowa T, Hastings IM, Molyneux ME, McCall PJ (2012). Entomological indices of malaria transmission in Chikhwawa district, Southern Malawi. Malar J.

[69] Mzilahowa T, Chiumia M, Mbewe RB, Uzalili VT, Luka-Banda M, Kutengule A (2016). Increasing insecticide resistance in *Anopheles funestus* and *Anopheles arabiensis* in Malawi, 2011–2015. Malar J.

[70] Wondji CS, Coleman M, Kleinschmidt I, Mzilahowa T, Irving H, Ndula M (2012). Impact of pyrethroid resistance on operational malaria control in Malawi. Proc Natl Acad Sci U S A.

[71] Riveron JM, Chiumia M, Menze BD, Barnes KG, Irving H, Ibrahim SS (2015). Rise of multiple insecticide resistance in *Anopheles funestus* in Malawi: a major concern for malaria vector control. Malar J.

[72] The Hunger Project: Epicenter strategy. www.thp.org/our-work/where-we-work/africa/epicenter-strategy. Accessed 29 Nov 2016 .

[73] Homan T, Di Pasquale A, Kiche I, Onoka K, Hiscox A, Mweresa C (2015). Innovative tools and OpenHDS for health and demographic surveillance on Rusinga Island, Kenya. BMC Res Notes.

[74] The Hunger Project: Our approach. www.thp.org/our-work/our-approach. Accessed 29 Nov 2016 .

[75] Wilson AL, Boelaert M, Kleinschmidt I, Pinder M, Scott TW, Tusting LS (2015). Evidence-based vector control? Improving the quality of vector control trials. Trends Parasitol.

[76] Guerra CA, Reiner RC, Perkins T, Lindsay SW, Midega JT, Brady OJ (2014). A global assembly of adult female mosquito mark-release-recapture data to inform the control of mosquito-borne pathogens. Parasit Vectors.

[77] Hiscox A, Otieno B, Kibet A, Mweresa CK, Omusula P, Geier M (2014). Development and optimization of the Suna trap as a tool for mosquito monitoring and control. Malar J.

[78] Mweresa CK, Omusula P, Otieno B, van Loon JJA, Takken W, Mukabana WR (2014). Molasses as a source of carbon dioxide for attracting the malaria mosquitoes *Anopheles gambiae* and *Anopheles funestus*. Malar J.

[79] Menger DJ, van Loon JJA, Takken W (2014). Assessing the efficacy of candidate mosquito repellents against the background of an attractive source that mimics a human host. Med Vet Entomol.

[80] Lines JD, Curtis C, Wilkes TJ, Njunwa K (1991). Monitoring human-biting mosquitoes (Diptera: Culicidae) in Tanzania with light-traps hung beside mosquito nets. Bull Entomol Res.

[81] Malaria Indicator Survey: Basic documentation for survey design and implementation. http://malariasurveys.org/toolkit.cfm. Accessed 29 Nov 2016.

[82] Roca-Feltrer A, Lalloo DG, Phiri K, Terlouw DJ (2012). Rolling Malaria Indicator Surveys (rMIS): A Potential District-Level Malaria Monitoring and Evaluation (M&E) Tool for Program Managers. Am J Trop Med Hyg.

[83] Chipeta M, Terlouw D, Phiri K, Diggle P. Inhibitory geostatistical designs for spatial prediction taking account of uncertain covariance structure. Environmetrics. 2016;28:e2425.

[84] Birley MH, Charlewood JD (1987). Sporozoite rate and malaria prevalence. Parasitol Today.

[85] Hay SI, Rogers DJ, Toomer JF, Snow RW (2000). Annual *Plasmodium falciparum* entomological inoculation rates (EIR) across Africa: literature survey, internet access and review. Trans R Soc Trop Med Hyg.

[86] Gimnig JE, Vulule JM, Lo TQ, Kamau L, Kolczak MS, Phillips-Howard PA (2003). Impact of permethrin-treated bed nets on entomologic indices in an area of intense year-round malaria transmission. Am J Trop Med Hyg.

[87] Diallo D, Doumbo OK, Plowe CV, Wellems TE, Emanuel EJ, Hurst SA (2005). Community permission for medical research in developing countries. Clin Infect Dis.

[88] World Health Organization (1999). Environmental Health Criteria 217: *Bacillus thuringiensis*.

[89] World Health Organization (2012). Handbook for Integrated Vector Management.

[90] Kabaghe AN, Chipeta MG, McCann RS, Phiri KS, Van Vugt M, Takken W (2017). Adaptive geostatistical sampling enables efficient identification of malaria hotspots in repeated cross-sectional surveys in rural Malawi. PLoS One.

