# Supplementary material for: Assessment of the effect of larval source management and house improvement on malaria transmission when added to standard malaria control strategies in southern Malawi: study protocol for a cluster-randomised controlled trial
Source: BMC Infect Dis. 2017 Sep 22;17:639. doi: 10.1186/s12879-017-2749-2 (PMC5610449; doi:10.1186/s12879-017-2749-2)
Supplement: Supplementary file 1 — World Health Organization Trial Registration Data Set for the MMP LSM/HI trial. (DOCX 117 kb) [file 12879_2017_2749_MOESM1_ESM.docx]

Additional file 1: World Health Organization Trial Registration Data Set for the MMP LSM/HI trial.

| **Data Category** | **Information** |
| --- | --- |
| Primary registry and trial identifying number | The Pan African Clinical Trials Registry  http://www.pactr.org/  PACTR201604001501493 |
| Date of registration in primary registry | 3 March 2016 |
| Secondary identifying numbers | Universal Trial Number: U1111-1179-7205  College of Medicine Research and Ethics Committee: P.05/15/1731 |
| Source(s) of monetary or financial support | Dioraphte Foundation, the Netherlands |
| Primary sponsor | Wageningen University |
| Secondary sponsor(s) | University of Malawi, College of Medicine |
| Contact for public queries | Willem Takken, willem.takken@wur.nl, +31 317 484652 |
| Contact for scientific queries | Robert McCann, robert.mccann@wur.nl, +265 994 292300 |
| Public title | Majete Malaria Project larval source management and housing improvement trial |
| Scientific title | Evaluation of the effect of larval source management and house improvement when added to an existing malaria control strategy (MMP LSM/HI trial) |
| Countries of recruitment | Malawi |
| Health condition(s) or problem(s) studied | Malaria |
| Intervention(s) | - Larval source management, including habitat modification and larviciding with *Bti* - Structural house improvement |
| Key inclusion and exclusion criteria | All households and residents in Majete Malaria Project catchment area are eligible for inclusion. |
| Study type | Cluster randomised controlled trial |
| Date of first enrolment | 1 May 2016 |
| Target sample size | 6480 |
| Recruitment status | Recruiting: participants are currently being recruited and enrolled |
| Primary outcome(s) | Entomological inoculation rate at the end of the trial |
| Key secondary outcome(s) | - Entomological inoculation rate at 2-month intervals over 2 years - Malaria vector community composition at 2-month intervals over 2 years - Malaria vector human blood index at 2-month intervals over 2 years - Peak malaria vector biting time at 2-month intervals over 2 years - Larval mosquito density at 2-month intervals over 2 years - Parasite prevalence in children aged 6-59 months at 2-month intervals over 2 years - Prevalence of anaemia in children aged 6-59 months at 2-month intervals over 2 years - Incidence of clinical malaria in children aged 6-59 months, measured in 2 cohorts followed for 1 year each |
